# Kinetic Oxidation Analysis in AISI 1045 Steel Using Infrared Thermography and Convolutional Neural Networks

**DOI:** 10.3390/ma19050920

**Published:** 2026-02-27

**Authors:** Oscar David Prieto-Sánchez, Antony Morales-Cervantes, Jorge Sergio Téllez-Martínez, Gerardo Marx Chávez-Campos, Edgar Guevara, Héctor Javier Vergara-Hernández

**Affiliations:** 1 División de Estudios de Posgrado e Investigación, TecNM-Instituto Tecnológico de Morelia, Maestría en Ciencias en Ingeniería Electrónica (MCIE), Av. Tecnológico 1500, Morelia 58120, Mexicoantony.mc@morelia.tecnm.mx (A.M.-C.); jorge.tm@morelia.tecnm.mx (J.S.T.-M.);; 2 Faculty of Science, Universidad Autónoma de San Luis Potosí, San Luis Potosí 78294, Mexico; edgar.guevara@uaslp.mx (E.G.)

**Keywords:** CNN, steel oxidation, infrared thermography, kinetic parameters

## Abstract

This study presents a pioneering approach, integrating infrared thermography and deep learning to analyse surface oxide layers on AISI 1045 steel, addressing the critical need for advanced monitoring in steelmaking processes. Using thermography for observation and semantic segmentation for accurate identification, 50 tests between 200 and 700 °C were analysed in a Joule-controlled heating system to study the formation and thickening of oxide layers on steel surfaces. A convolutional neural network (CNN), specifically SegNet, was trained for semantic segmentation, facilitating detailed analysis. The model achieved an overall accuracy of 96.40% in identifying the presence of oxide. By quantifying pixelation changes, relationships in oxide evolution kinetics were obtained, and by quantifying the activation energy in isothermal cases, the magnitude is in the range reported by other works. The approach also highlighted the potential for non-destructive monitoring and control on a large scale without compromising personnel safety. This potential could improve industrial process control, predict surface quality or provide data relevant to sub-processes.

## 1. Introduction

In recent years, steel production and demand have increased considerably, driving the optimisation of processes to produce higher-quality products [1,2]. Elevating the metal to sufficiently high temperatures induces the formation of a surface oxide film, thereby modifying both its microstructure and associated material properties [3]. Therefore, while high-temperature heat treatments improve of steel quality and mechanical properties, they can also lead to oxidation and decarburization, posing significant challenges for steel plants [4]. Factors such as heating time, atmospheric oxygen levels, and the carbon content of the steel are crucial in determining the extent of oxidation [5,6]. Despite technological advances in steel manufacturing, high-temperature surface oxidation remains a predominant issue. This oxidation results in significant material losses during critical stages such as reheating of semi-finished products and rolling, with estimated losses of up to 10% of the total material. In cases where oxidation exceeds acceptable levels, the material is often rejected, leading to even greater economic losses [7,8]. The development of automated methodologies for material identification and defect detection during steel handling is crucial, as these approaches enhance final product quality while reducing damage and material waste [4,9]. At present, the quality assessment of steel products is predominantly performed using optical–digital systems that capture and process images of their surfaces [10]. Various algorithms for assessing product quality are constantly updated [11,12]. However, there is a need for greater generalisation and description of the specific process characteristics that account for the emergence of defects arising from the material’s specific morphological characteristics [13]. Therefore, this research is dedicated to detecting oxide scale in thermographic images using Convolutional Neural Networks (CNNs) and semantic segmentation. An approach that can be used in future studies to identify oxidation kinetics (as evidenced by thickening of the oxide layer) or imperfections that may develop during rolling operations [14].

Several advanced analytical techniques, such as scanning electron microscopy (SEM), transmission electron microscopy (TEM), X-ray diffraction (XRD), focused ion beam (FIB), energy-dispersive X-ray spectroscopy (EDX), and atomic force microscopy (AFM), have been implemented for the characterisation and determination of chemical transformation mechanisms. Through these techniques, it is possible to determine nodular or whisker-like morphologies. As more oxide forms, the products change in size. Therefore, both their morphology and size confer specific optical properties on the structures at high temperatures. In this context, by applying Planck’s law and geometric optics, it is proposed that the degree of change in reflection and radiation characteristics serves as an indirect means of observing changes in the thickness of the oxide layer. Digital analysis of thermographs can offer a non-invasive, efficient alternative for real-time processes. In this work, a controlled experimental procedure was implemented to correlate the evolution of pixel counts on a sample surface with the kinetics of oxide layer formation. In addition, the characteristics of the capture, control, and processing of the thermographs are established to explain the distribution phenomenologies.

Prior investigations indicate that Support Vector Machines (SVMs) and Artificial Neural Networks (ANNs) are the predominant approaches for predicting material properties, owing to their effectiveness in modelling complex nonlinear relationships [15]. Existing literature primarily addresses the detection of surface defects in metals, which often differ in geometry, scale, and texture. Nevertheless, this type of quantitative analysis has not been explored using thermographic imagery, particularly for the processing and identification of oxidation in steel at elevated temperatures. Consequently, the scope of the present study provides an appropriate framework for examining and implementing image processing techniques in this domain.

Several studies have reported the use of Convolutional Neural Networks (CNNs) for applications in this domain. In [16], a cascaded autoencoder architecture was implemented to identify damaged pixels and determine defect categories, achieving an accuracy of 89.6%, which meets the reliability and precision criteria for metal defect detection. In [12], a U-Net–based framework was introduced for detecting crack widths on concrete wall surfaces, incorporating image pre-processing and post-processing of the network output, and achieved an accuracy greater than 95%. Similarly, [13] presented an automated defect recognition approach for steel manufacturing based on a CNN-driven U-Net for semantic segmentation, reporting an accuracy of 91.5%. Finally, ref. [14] employed a ResNet18 CNN for semantic segmentation, designed to extract and integrate multiple defect-related features, yielding an efficiency of 86.2%.

For the purpose of detecting metal defects, various neural network topologies and training methods have been employed. Nevertheless, thermographic image processing was not used in any of the experiments. Controlling oxidation and decarburization during heat treatments can be aided by examining these processes. The main contribution of this study is the use of CNNs and semantic segmentation to identify steel surfaces and oxide scale in thermographic images, pixel by pixel, enabling a more precise quantitative assessment of oxidation levels.

## 2. Materials and Methods

### 2.1. Overview of the System

This study integrates infrared thermography and semantic segmentation techniques using convolutional neural networks (CNNs) to analyse the formation and thickening of oxide layers on AISI 1045 steel at high temperature under conditions of a predominant oxygen concentration in the laboratory room where the tests were carried out. In particular, for this work, the room was kept closed to avoid drafts and to create an experimental environment with natural convection. The system is composed of a controlled Joule heating setup that allows precise temperature regulation using the impressed current. This is essential for observing oxide layer development on the material’s surface and for exposing the sample to still air under normal ambient oxygen conditions.

The process begins by capturing thermal images at various stages of oxidation to track and quantify oxide-scale evolution under real isothermal conditions. Maintaining a constant current flow keeps the energy developed constant by keeping the voltage difference constant during the heating process [2]. Therefore, reproducibility of the temperature index across samples is easily achievable, ensuring that the experimental conditions for different samples are consistent with the results in terms of the amount of oxide formed.

### 2.2. Experimental Setup

**Figure 1:**
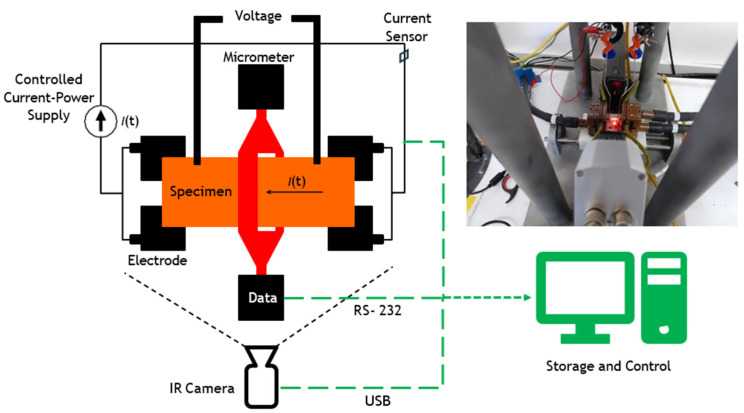
Configuration of the experiment for the acquisition of images by infrared thermography. The green dashed line indicates the data flow from the recording devices to the processing unit.

Fifty high-temperature oxidation experiments were carried out using cylindrical specimens of AISI 1045 steel, measuring 26 mm in length and 3.8 mm in diameter. The specimens were machined with enlarged grip heads to ensure secure electrical contact. The experiments aimed to investigate the surface changes induced by forming oxide layers in a controlled environment. For this, a specifically designed Joule system is used, which allows the control of both the temperature and the current supplied to the samples. Each experiment followed a gradual heating sequence, increasing the current in steps of 16 amps per minute up to 160 amps, holding it constant for 30 min to facilitate oxide layer formation and allow analysis of surface changes. Subsequently, controlled cooling was carried out, reducing the current in steps of 16 A/min until reaching 0 A. During the entire process, 50 thermographic images per experiment were captured to visualise the surface temperature distribution, resulting in a total of 2500 distinct images across all experiments. The data obtained, including thermographic images, temperature and current measurements, were stored in a database. The experimental setup for acquiring infrared images during heating 1045 steel is shown in Figure 1. The IR camera and specimen were kept in a fixed position throughout all experiments to ensure a consistent field of view and minimise geometric variability. Voltage was measured using metallic contacts located near the clamping region and outside the segmentation ROI. Any contact-related radiometric artefacts (vertical thermal features) are excluded from the gauge section analysed by the CNN, thus preventing bias in the oxide masks and kinetic descriptors.

It should be noted that the Joule heating protocol was current-controlled, and therefore, the maximum temperature reached during the holding stage could vary slightly among specimens due to differences in electrical resistance, surface condition, and heat dissipation. In this study, most experiments reached steady-state peak temperatures around 700 °C, while a limited number of runs reached values close to 800 °C under the same current profile. For the Arrhenius-type analysis, representative experiments were selected based on the measured steady-state temperature during the isothermal holding period, following conventional practice where multiple kinetic constants are evaluated across different temperature conditions. The selected temperature window (200–700 °C) was chosen to enable comparison with oxidation kinetics trends reported in the literature for low-carbon steels. Importantly, unlike postmortem approaches that require interrupting the test for cross-sectional or gravimetric measurements, the proposed thermographic monitoring provides continuous, in situ tracking of oxidation-related surface evolution throughout the heating, holding, and cooling stages.

### 2.3. Image Acquisition and Pre-Processing

The capture of images of the material has been taken with the factory-calibrated Optris digital infrared camera PI 1M, (Optris GmbH & Co. KG, Berlin; Germany), which is based on a microbolometer focal plane arrangement, with thermal sensitivity of <1 K (700 °C), <2 K (1000 °C), an accuracy of ±1%, a spectral range of 0.85–1.1 μm and a temperature range of 450–1800 °C. This thermal imaging system provides real-time thermal information with a resolution of 764 × 480 pixels that can be saved in digital format. Measurements were made at a constant emissivity of 0.8, recommended for polished steel with oxide scale [17]. No additional temperature-sensing instrumentation was implemented to avoid interference. It is worth noting that the specific use of a short-wavelength sensor significantly minimises measurement errors associated with emissivity variations compared to standard long-wave thermography. Recent studies [18,19] have validated the use of infrared thermography as a robust tool for detecting oxide layer formation on AISI 1045 steel. These works demonstrated that the strong emissivity contrast between the steel substrate and the growing oxide scale, captured as thermal anomalies, correlates with the physical presence of oxide domains, achieving segmentation accuracies exceeding 96% when compared to expert visual inspection. This establishes the physical basis for using thermographic pixel data as a validated surface proxy for training deep learning models. Therefore, the kinetic parameters reported in this study should be interpreted as apparent surface-coverage descriptors that may include combined contributions from oxidation progression and emissivity evolution during high-temperature exposure. Future work will incorporate thermocouple-assisted validation to further verify absolute temperature readings and refine emissivity settings during high-temperature exposure, strengthening traceability for thermographic temperature measurements.

Pre-processing involved standardising the format and size (382 × 288 pixels) of the thermographic images and normalising them to improve the performance of the CNN. The data were split into training and test sets at an 80–20% ratio, chosen at random. The training set comprised 2000 thermal images with a resolution of 382 × 288, each annotated to differentiate the specimen, the oxide layer, and the image background. An additional 500 thermal images were allocated to the test set. While the training data were used to learn and extract underlying patterns, the test data were used to evaluate the performance of the network.

### 2.4. CNN Training and Segmentation

**Figure 2:**
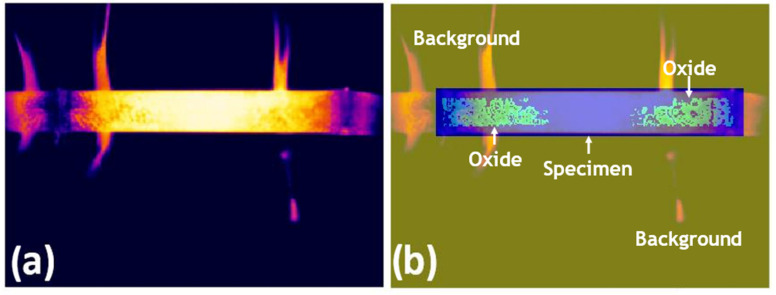
(**a**) Original infrared thermography image, the rainbow-type temperature scale shows white as the highest temperature, followed by the colours: yellow, orange, red, pink, violet, blue, and black, respectively. and (**b**) corresponding pixel-wise label overlay illustrating the background, oxide layer in green, and specimen regions in blue.

For CNN-based semantic segmentation, pixel-wise annotation was applied to the training images, assigning labels to the background, oxide layer, and steel specimen. Manual pixel-level annotations were generated by a trained operator with expertise in infrared thermography and high-temperature oxidation processes. The labelling followed consistent criteria based on persistent thermal contrast, spatial continuity, and temporal coherence of oxidised regions across consecutive frames. Pixel labelling was performed by assigning class identifiers to each region: oxide (label 3), specimen area (label 2), and background (label 1). These categorical values were used to generate pixel-wise masks for CNN training. A pixel-labelled image is one in which each pixel value corresponds to the categorical label of that pixel. Figure 2 shows an example of a pixel-labelled thermogram for AISI 1045 steel.

A SegNet architecture, derived from a standard convolutional neural network (CNN) framework [20], was employed.

The selection of SegNet over other architectures, such as U-Net or ResNet, was motivated by several key characteristics that make SegNet particularly suitable for this task. First, the encoder–decoder architecture of SegNet uses max pooling indices to store information about feature locations during downsampling. This allows it to preserve spatial information more effectively than other networks. This is crucial for our application, where precise boundary delineation of oxide layers is essential for accurately tracking oxidation kinetics.

Another reason for choosing SegNet is its computational efficiency compared to deeper networks like ResNet. Since our dataset is relatively small, using a deeper network could lead to overfitting and increased computational costs. SegNet strikes a good balance between accuracy and efficiency, making it particularly suitable for the real-time processing requirements of the proposed industrial monitoring framework, where computational efficiency is as critical as segmentation accuracy.

While U-Net is a strong candidate for image segmentation, it is often preferred in tasks that require high precision in detecting very small objects, such as in biomedical imaging. In contrast, our study involves segmenting relatively large regions, such as oxide layers and steel surfaces, where maintaining spatial coherence across the entire image is crucial.

**Figure 3:**
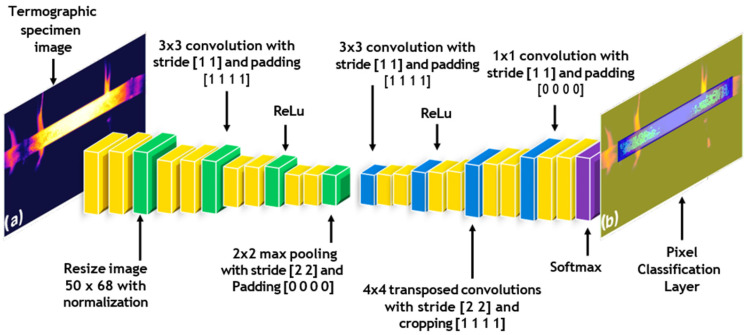
Convolutional Neural Network (CNN) Layer Diagram for Semantic Segmentation in Thermograms: From Image-Related Function Application to Spatial Reduction and Subsequent Unpooling. (**a**) Specimen thermogram, and (**b**) spatial segmentation image.

Figure 3 shows the representation of a CNN and its inverse implementation, which was added to enable pixel-by-pixel classification in the image. The upsampling process was performed the same number of times as the downsampling process to ensure that the final image was the same size as the input image. The output image preserved the same spatial dimensions as the input, a requirement for semantic segmentation. Accordingly, the network was trained to assign each pixel to one of three classes (background, specimen, or oxide). In the final network layer, these classes are visualised with the oxide represented in light green, the specimen in blue, and the background in yellow.

### 2.5. Post-Processing and Analysis

Post-processing involved analysing changes in pixel distribution in the thermographic images to quantify the kinetics of oxide layer growth. The data obtained from the SegNet model were analysed to determine key parameters, such as the activation energy of the oxidation process under isothermal conditions. This method allowed for a more precise quantification of the oxide layer growth, correlating pixelation changes with the kinetics of scale formation.

The thermal images were downsampled to 50 × 68 pixels in order to limit the number of trainable parameters. The implemented SegNet model comprises a 10-layer sequence and adopts a semantic segmentation architecture based on successive downsampling and upsampling operations.

SegNet is a deep learning architecture specifically designed for image segmentation tasks. It follows an encoder–decoder structure, where the encoder is a typical Convolutional Neural Network (CNN) that reduces the spatial resolution of the input image by applying convolutional layers followed by pooling operations. These operations help capture higher-level features in the image, such as edges and textures, while also reducing the computational complexity by lowering the dimensionality of the image.

The decoder part of SegNet is responsible for restoring the spatial resolution of the image, which is critical for pixel-level classification tasks such as semantic segmentation. The decoder performs “upsampling,” where the spatial resolution is gradually restored to its original size by reversing the pooling operations performed in the encoder. SegNet uses a specific technique called max-pooling indices to store information about where the maximum activation occurred during the downsampling phase. This helps in accurately reconstructing the image during upsampling, as it reuses the location information of key features.

In our implementation, SegNet was trained to classify each pixel into one of three categories: background, oxide, and steel specimen. This pixel-wise classification allows us to identify and quantify the presence of oxide layers on the steel surface. To evaluate the performance of the network, metrics such as Global Accuracy, Mean Intersection over Union (Mean IoU), and Boundary F1 Score (Mean BFScore) were used. These metrics are essential in assessing how well the network distinguishes between the three classes, especially in challenging areas where the oxide and steel boundaries may be blurred or incomplete.

One key advantage of SegNet over other architectures is its ability to maintain boundary details during segmentation. In tasks such as ours, where precise localisation of oxide layers is crucial for studying oxidation kinetics, preserving boundary information directly improves the reliability of the analysis. Additionally, the encoder–decoder architecture of SegNet enables efficient training with relatively small datasets, which is beneficial when thermographic data is limited.

**Figure 4:**
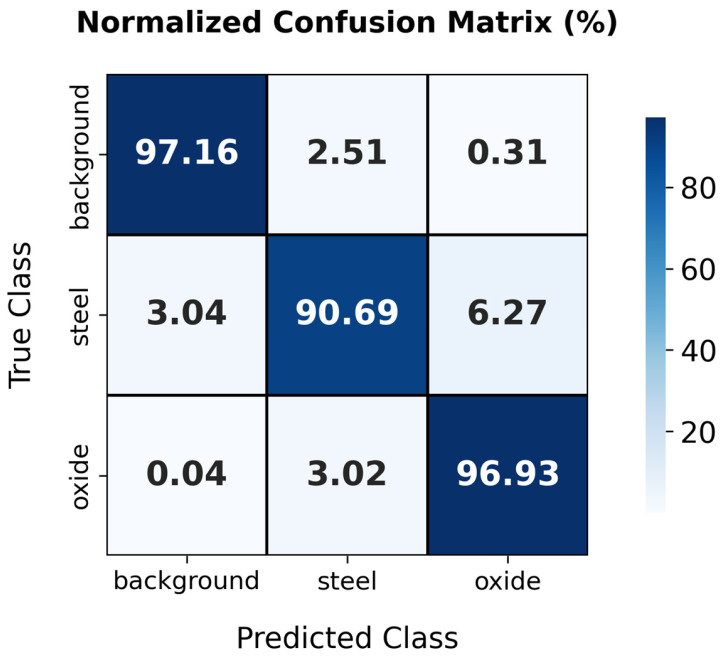
Confusion matrix demonstrating the classification performance of the SegNet model, with high accuracy in oxide scale detection and minor misclassifications between steel and oxide scale.

The processing pipeline and network layers of the implemented CNN are illustrated in Figure 3. The model was trained for 200 epochs, resulting in the overall loss function converging to a plateau. An epoch corresponds to a complete forward and backward pass through the entire training dataset. Class weight was balanced to avoid bias in favour of the ruling class. To ensure reproducibility, network training was performed with a fixed random seed (seed = 42), the SGDM optimiser, and a constant learning rate of 0.001. The metrics used to evaluate the proposed SegNet are: Global Accuracy, which represents the percentage of correctly classified pixels across all classes; Mean Accuracy, which calcu- lates the average accuracy per class to ensure balanced performance across background, oxide, and specimen; Mean Intersection over Union (Mean IoU), a measure of the overlap between the predicted segmentation and the ground truth, averaged across all classes; Weighted IoU, which is similar to Mean IoU but assigns greater weight to larger classes, ensuring that dominant classes like steel and oxide have a higher impact; and Mean BFScore, or Boundary F1 Score, which evaluates the accuracy of boundary detection, ensuring precise delineation between regions. Beyond these metrics, the performance of the SegNet model was further evaluated using a confusion matrix (see Figure 4). This provides a detailed representation of classification outcomes by reporting correct and incorrect predictions for each class, including background, oxide, and specimen. This helps identify patterns of misclassification and provides insights into how the model confuses certain classes, such as misclassifying oxide as steel or vice versa.

## 3. Results and Discussion

The sensitivity of individual pixel classifications to image preprocessing and threshold selection is an inherent characteristic of thermographic image analysis. In the present study, the spatial downsampling applied prior to CNN training and analysis was performed consistently for both images and labels. Since the kinetic analysis is based on normalised surface coverage fractions and their temporal evolution, the reduction in spatial resolution does not affect the extracted kinetic trends, but only the absolute pixel count. Consequently, variations in brightness thresholds or preprocessing parameters primarily affect instantaneous pixel counts, but do not alter the overall time-dependent trends used to extract apparent kinetic parameters. All quantitative kinetic results reported in this work are based on CNN-derived semantic segmentation masks, while the brightness sweep analysis is provided solely for qualitative visualisation.

Table 1 summarises the classification metrics of the proposed network, offering a quantitative evaluation of its ability to discriminate steel specimens, oxide regions, and background in thermographic imagery. Overall prediction accuracy is defined as the proportion of correctly classified pixels across all three classes within the test dataset. The CNN was then applied to the images to analyse the resulting segmentation performance. To implement the network, we utilised the MATLAB V2024b Deep Learning Toolbox (MathWorks, Natick, MA, USA) [21] for its flexibility in designing and training convolutional neural networks. The SegNet architecture was trained with the Adam optimiser, using a learning rate of 10−4 and a batch size of 16. We conducted experiments to compare different network configurations, such as varying the number of layers and filter sizes, but found that the 10-layer architecture provided the best trade-off between accuracy and computational efficiency. Additionally, we experimented with different optimisers, including SGD and RMSProp, which are commonly used for training neural networks such as SegNet. However, Adam consistently achieved faster convergence and higher final accuracy, making it the most suitable choice for this task and dataset. These outputs were compared with the corresponding ground-truth labels to determine the number of pixels correctly classified as background or specimen.

The evaluation of the proposed model, which classifies background, oxide scale, and specimen in thermographic images, yielded encouraging results. A Global Accuracy of 96.40% demonstrates the overall high efficiency of the model in correctly classifying pixels. The Mean Accuracy reached 94.93%, showing consistent and balanced performance across the different classes. The Mean Intersection over Union (Mean IoU) was 83.29%, highlighting the ability of the model to identify and outline areas of interest in the images accurately. The Weighted IoU stood at 93.58%, indicating particular effectiveness in segmenting the more prevalent classes. Lastly, the Mean BFScore of 81.38% underscores the proficiency of the model in detecting object boundaries, which is essential for applications where accurate delineation of elements is critical. These results demonstrate the high precision and adaptability of the model in thermographic image segmentation. One challenge observed during the segmentation process was the effect of misclassified pixels at the boundaries between oxide and steel. These errors, while not significantly affecting overall accuracy, did result in slight reductions in boundary-detection precision, as reflected in the Mean BFScore. For instance, regions with irregular shapes or lower thermal contrast between oxide and steel exhibited higher pixel misclassification rates. These errors mainly impacted the segmentation of small or thin oxide layers, where the model occasionally confused them with the background. Future work could focus on refining the boundary detection process or applying post-processing techniques, such as conditional random fields (CRF), to improve edge classification.

Although SegNet played an essential role in segmenting thermographic images, the principal contribution of this study lies in applying CNN-based semantic segmentation to analyse oxidation kinetics, particularly in the context of infrared thermography for non-destructive testing. The ability to quantify oxidation layer growth through pixel-wise classification offers a novel approach for industrial applications, enabling more accurate real-time monitoring of material degradation. This makes the approach more relevant to industries seeking reliable, non-invasive methods for steel quality control. It is important to emphasise that the oxide evolution analysed in this work is derived from a two-dimensional, thermography-based surface proxy obtained via CNN semantic segmentation. The extracted pixel-based metrics represent the evolution of oxide-covered surface area and associated thermal anomalies [18,19], rather than a direct measurement of oxide thickness or mass gain. Consequently, the kinetic parameters reported herein should be interpreted as apparent surface-coverage descriptors, suitable for non-contact monitoring and comparative analysis, but not as absolute oxidation kinetics in the classical gravimetric sense.

Figure 4 presents the confusion matrix produced by the SegNet model, averaged across ten repeated runs, which is consistent with the statistical outcomes summarised in Table 1.

The confusion matrix details the performance of the model in classifying background, steel, and oxide scale in thermographic images. For the background, a notable 97.16% of the pixels were correctly identified, but the model confused 2.51% of steel pixels and 0.31% of oxide scale pixels as background. In the steel classification, the model was accurate in 90.69% of cases but erroneously identified 3.04% of background pixels and 6.27% of oxide-scale pixels as steel. The oxide scale category showed high precision (96.93% accuracy), although small proportions of background (0.04%) and steel (3.02%) were misclassified as oxide scale. These results reflect a high degree of accuracy in classifying background and oxide scale, with slightly lower efficiency in identifying steel. This particularly highlights the tendency of the model to confuse steel with oxide scale and vice versa, which is a crucial aspect for future improvements to the model.

In thermal image segmentation, overall test accuracy is a crucial metric for evaluating model performance. The overall accuracy, calculated as the percentage of correctly classified pixels in the entire test dataset, reflects the overall effectiveness of the model in assigning the correct category (background, oxide scale, or sample) to each pixel.

The global accuracy is given by:

(1) $$Global\;Accuracy=\frac{Number\;of\;correct\;predictions\;pixels}{Total\;number\;of\;samples\;pixels}$$

**Figure 5:**
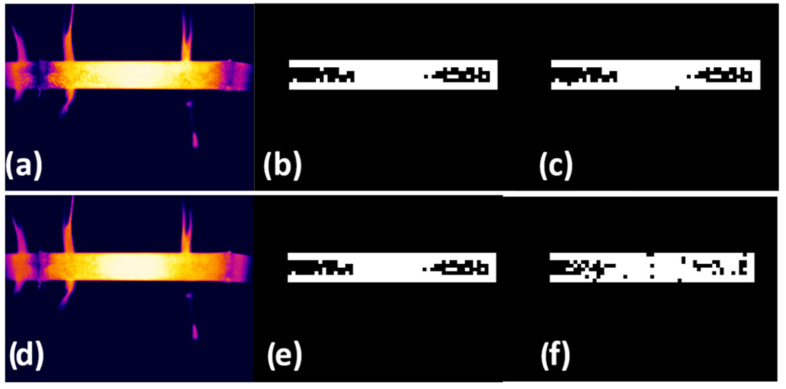
Best IoU (95.17%): (**a**) test infrared thermography image, (**b**) mask label and (**c**) predicted label; worst IoU (75.54%): (**d**) test infrared thermography image, (**e**) mask label and (**f**) predicted label. Again, on the rainbow-type temperature scale, white represents the highest temperature, followed by the colours: yellow, orange, red, pink, violet, blue, and black, respectively. The mask label and predicted label are optimally displayed in black and white.

To calculate this metric, the total number of correctly classified pixels, meaning those for which the predicted classification of the model matched their actual category in the test data, was divided by the total number of pixels in the test dataset. Finally, the result was multiplied by 100 to express it as a percentage. This approach provides a comprehensive view of the accuracy of the model, encompassing all categories in the dataset and offering a precise measure of performance in a controlled test environment. In this study, the global accuracy reached 96.40%, indicating a high level of precision in the segmentation of thermal images by the model. Figure 5. Examples of segmentation performance showing the highest IoU value (95.17%), with (a) the test image, (b) the corresponding ground-truth mask, and (c) the predicted output, as well as the lowest IoU value (75.54%), illustrated by (d) the test image, (e) the ground-truth mask, and (f) the predicted output. This technique accurately identifies irregularly shaped objects by labelling individual pixels.

Although the semantic segmentation of the frames was performed in MATLAB, the Fiji distribution of ImageJ 1.54g (NIH, Bethesda, MD, USA) [22] was used to clearly visualise the detected masks. Applying advanced visualisation tools in Fiji highlights oxidation patterns, allowing for a better appreciation of the changes identified by the SegNet model. This process substantially supports the analysis of segmentation results, ensuring that visual representations are closely aligned with the semantic segmentation process and provide a clearer understanding of model performance.

**Figure 6:**
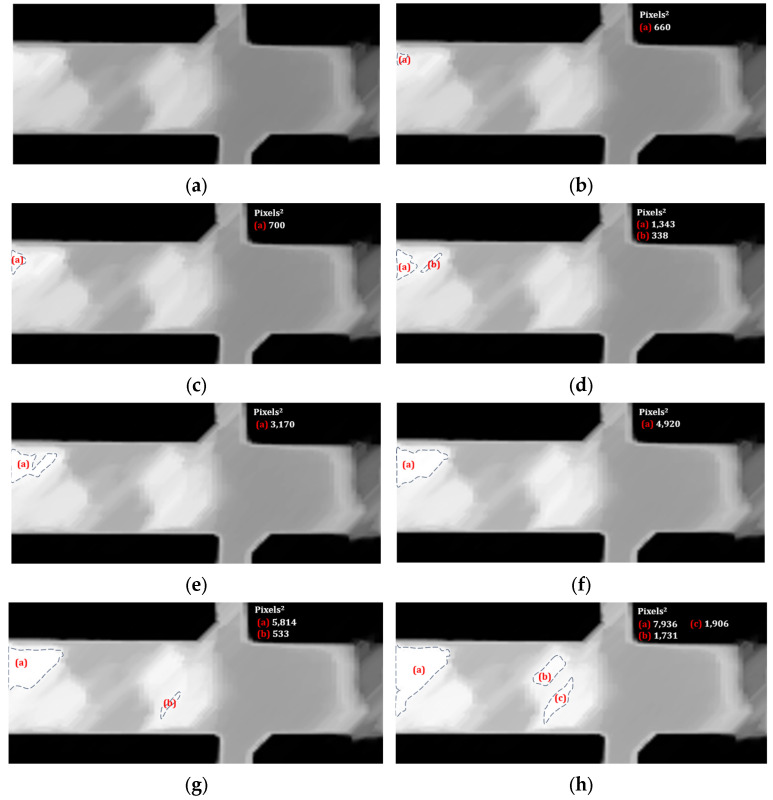

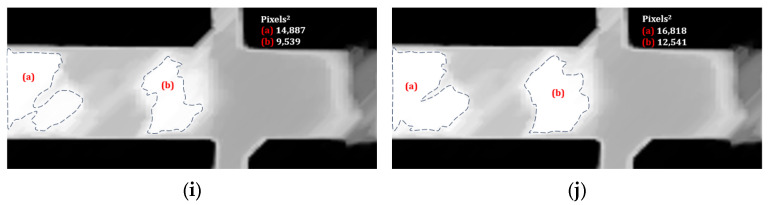
Apply brightness intensity (%) to a test thermogram in greyscale at 22 min and 39 s, to identify the qualitative mechanism of oxide layer coverage and thickening in high-reflection areas with contours that are segmented lines whose regions are identified with (a), (b), and (c) in red, and their corresponding magnitude in Pixels^2^. The brightness in the subfigures was specified as percentages (**a**) 0%; (**b**) 6%; (**c**) 8%; (**d**) 10%; (**e**) 12%; (**f**) 14%; (**g**) 16%; (**h**) 18%; (**i**) 20%; (**j**) 22%.

Under steady-state conditions during a test, at approximately 22 min and 39 s, a frame was obtained in which a grayscale transformation was performed. The partial image result shown in Figure 6a indicates that the macroscopically observed oxide layer did not form homogeneously over the entire surface.

A diffuse surface effect could explain the differences in hue caused by high roughness, which is a product of the morphologies of different oxide structures. In this regard, stable whiskers and compact granule-like morphologies for the same type of oxide were reported by Jung-Yeul et al. [23] at temperatures of 500 °C and 700 °C, respectively. In addition, Falk and Falk [24] reported that the degree of reflectivity decreases as the amount of oxide on the surface increases. Another preferential aspect of formation mentioned by Deng et al. [25] is that by determining the surface topology of HSS steel using advanced techniques such as X-ray diffraction (XRD), focused ion beam techniques and scanning electron microscopy, they found that around pre-existing metal carbides, the oxide formation is promoted more rapidly than in other carbide-free zones. In other findings, using atomic force microscopy (AFM) to monitor the oxidation process over time, they observed that the increase in arithmetic mean roughness is attributable to the thickening of the oxide layer. They proposed that growth can be modelled using a parabolic equation.

The digital analysis was continued in Fiji to gradually increase the brightness (luminosity) by 2% in Figure 6a over a region of the specimen exhibiting high contrast. The application of brightness was used to demonstrate that the thickening of the oxide layer manifests as a darkening of the region.

A relevant change in the image occurred at 6% brightness (Figure 6b), as alterations showing white-coloured regions were detected. Suppose the white-coloured areas were strategically associated with a thin oxide layer. In that case, a pattern of disappearing oxide scale on the surface can be created sequentially, as shown in the remaining images from Figure 6c (8% brightness) to Figure 6j (22% brightness). As shown in each image, the growing white areas were outlined with segmented lines, and the surface area in Pixels^2^ was calculated using a Fiji function to illustrate the orientation of their local formation, due to the preferential initial origins of oxide evolution.

Additionally, the areas were qualitatively coloured via image superposition to simulate the growth and union mechanism of oxide-layer thickening at a specific point in time during the process, as observed in the reverse image sequence from Figure 6j to Figure 6a. Table 2 lists the magnitudes of the surfaces in Pixels^2^ estimated for each image in Figure 6, for each clear region (left and right). The colours used to identify them are also specified.

**Figure 7:**
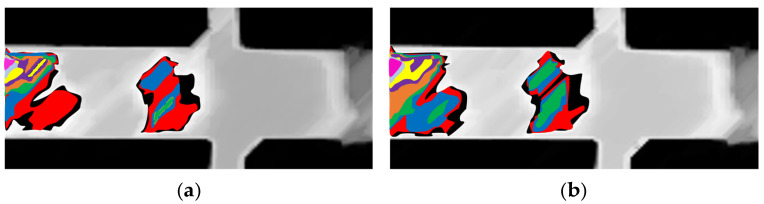
Overlay of oxide coverage areas with colours based on brightness intensity application analysis for two instances of the isothermal test in grayscale with: (**a**) 22% initial brightness (22 min and 39 s); (**b**) 32% initial brightness (22 min and 59 s). The areas are reduced according to the following colour scheme: Black, red, dark blue, green, orange, purple, yellow, light blue, and pink.

Figure 7a superimposes the coloured areas, indicating the oxide formation process on the initial substrate, with the frame obtained at 22 min and 39 s used as the reference. Although it is considered that, after forming an oxide layer in a pattern on other regions of the plate surface, only its thickness increases, a second frame obtained at 22 min and 59 s was analysed using the same strategy. Figure 7b shows the pattern of superimposed images with coloured areas that are very similar to those obtained from the frame 20 s earlier. In this case, the intensity and brightness percentages were modified: 9% was required to capture the first white region in the image, and the brightness increased by 3% to 32%. This indicates that as the oxide layer increases, the ability to shine increases as well.

**Figure 8:**
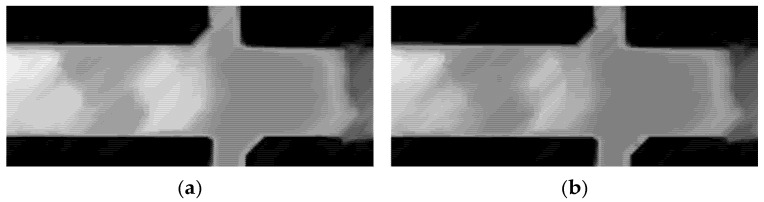
Grayscale thermal frames at two different times indicate darkening due to increased thickness: (**a**) 22 min and 39 s; (**b**) 24 min and 5 s.

The patterns in Figure 7 appear very similar. However, slight differences in the profile are observed in the sequence, which are attributed to the increased thickness of the oxide layer. By comparing frames from the 22 min 39 s and 24 min 5 s time points (2 min 34 s apart), it is evident that the surface darkens, consistent with low emission, which is hypothetically associated with the thickening of the oxide layer. The grayscale frames are shown in Figure 8.

Although the actual three-dimensional thickening mechanism is complex, it is assumed that the point-thickening rate of the oxide layer is proportional to the instantaneous local densification rate and inversely proportional to the pixelation, if the model used by Burke and Shiau [26] is adopted for analysis. Then, it is possible to provide a mathematical basis for parameterising the oxide-layer thickening suggested by Deng et al. [25]. According to the above, it is proposed that:

(2) $$\frac{dP}{dt}=K\cdot \frac{1}{P};\;\;\left[\frac{Pixels}{s}\right]=\left[\frac{{Pixels}^{2}}{s}\cdot \frac{1}{Pixels}\right]$$

The solution of Equation (2) emulates the parabolic law of grain growth in the crystallographic microstructure of a metallic material [27], where, in context, K represents the kinetic parameter and measures the rate of change in the variable of interest during the process. Similarly, in this study, P is the variable quantifying pixelation, referring to the degree of relatively punctual pixelation in the thermal image.

(3) $$P^{2}\cdot P_{0}^{2}=K\cdot t$$

The solution to Equation (2), given in Equation (3), indicates that the initial state of the pixelation characteristic of the steel substrate is evaluated as zero. Substituting $P_{0}^{2}$ and applying natural logarithm to the terms of Equations (3) and (4) is obtained. In the present framework, the evolution of the oxidised surface area, quantified here by the normalised oxide coverage proxy P, follows a phenomenological relationship analogous to the parabolic rate law, as evidenced by the observed thickening of the oxide layer over time. The resulting kinetic parameters are thus reported as apparent surface-coverage descriptors suitable for comparative monitoring and Arrhenius-type analysis.

(4) $$ln\left(P\right)=\frac{1}{2}ln\left(K\right)+\frac{1}{2}ln\left(t\right)$$

**Figure 9:**
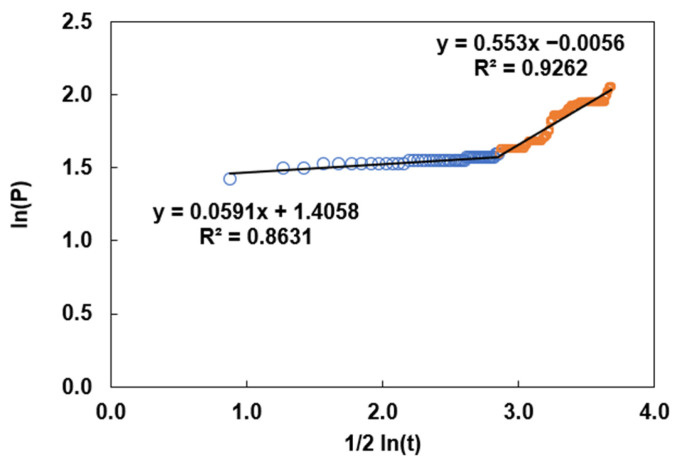
Data with linear trend functions for determining the kinetic parameter K from a pixelated history recorded at a point on the sample surface. The empty blue and orange circles indicate the data range that determines the best correlation coefficient for fitting the linear model in the spline specification.

Data from CNN-based semantic segmentation at different sampling points in experimental tests can be fit to the linear model in Equation (4). In some cases, more than one equation was required to determine the trend, as shown in Figure 9.

Therefore, the kinetic parameter K associated with the ordinate data was quantified for each expression. The trend of the straight lines represents a change in pixelation associated with a change in oxide thickness under isothermal process conditions.

(5) $$K=Ae^{\left(-\frac{Q}{RT}\right)}$$

**Figure 10:**
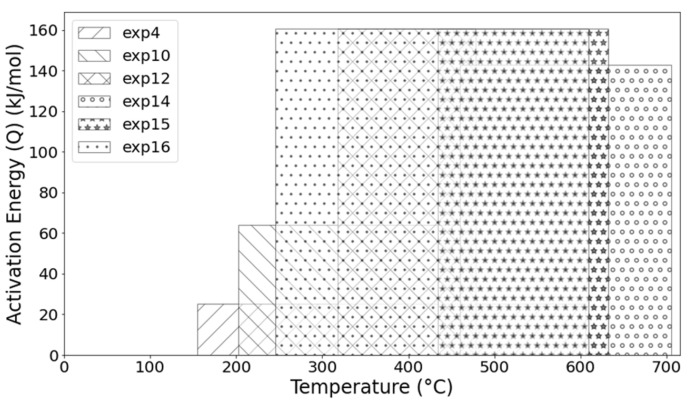
Magnitude of the activation energies (Q) of the evolution of oxide layers obtained from analysing processes at different temperatures.

Equation (5), known as the Arrhenius equation, is a model for interpreting the influence of temperature on reaction rates, as cited by Leidler [28]. In this case, the kinetic parameter K is given by an exponential function, where Q denotes the activation energy for the pixelation change associated with oxide thickening, R is the universal gas constant, and T is the temperature in Kelvin. The parameter A is known as the frequency factor, interpreted here as the frequency of pixelation changes associated with the oxide-formation reaction. Leidler notes in the rate-temperature data analysis that A and Q span a wide range of temperatures and can therefore often be treated as independent of temperature. It is evident that both phenomena, the change in pixelation and the thickening of the oxide layer, can be modelled by the parabolic law. Therefore, it would be practical to relate both changes by measuring the thickening rate, given the incremental trend indicated by the positive slope in the equations of Figure 9. Furthermore, as evidenced by the change in slope, it is possible to determine whether a chemical change, a physical change, or both occur simultaneously in the constitution and characteristics of the oxide layer (which is beyond the scope of this work). Data processing for the activation energy calculation is evident in the bar graph in Figure 10, where the bars overlap across broad temperature ranges. As shown, the magnitude for processes between 600 °C and 700 °C in dry gas is between 140 kJ mol^−1^ and 160 kJ mol^−1^, very close to the value of 168.27 kJ mol^−1^ reported by Jung-Yeul et al. [23]. Although slight variations in activation energy were observed across the tested temperatures, these fluctuations can be attributed to the complex, concurrent growth kinetics of multiple oxide phases (hematite, magnetite, and wustite) characteristic of commercial AISI 1045 steel. Consequently, the reported values (140–160 kJ mol^−1^) should be interpreted as the effective activation energy for the overall surface-coverage process. The consistency of this magnitude across the range supports the experimental scatter of the present dataset. As shown in Figure 10, a trend in pixelation change during oxide-layer thickening over the range 300 °C to 650 °C, obtained from three independent experiments, indicates a constant activation energy.

This study demonstrates the effectiveness of implementing a thermal image segmentation model. One of the main challenges encountered was the computational time required to analyse thermal images due to their complexity and high resolution. However, once the fundamental aspects of the specific methodology were determined, it could be applied to real-time scenarios, as it offers greater accuracy than alternative object detection methods [29].

Direct post-mortem microstructural characterisation techniques, such as SEM cross-sectional analysis or oxide thickness measurements, were not performed in the present study. To support physical verification, optical macrographs of representative cooled specimens are provided in the Supplementary Material (Figure S1), showing oxidation traces whose spatial location and morphology qualitatively correspond to the oxidation regions detected by infrared thermography and segmented by the CNN during high-temperature exposure. It should be noted that, after cooling, these oxidation features may appear visually subtle in optical images, whereas infrared thermography yields significantly greater radiometric contrast due to emissivity changes at elevated temperatures. This highlights the advantage of the proposed thermographic CNN approach over conventional visual inspection. While such techniques provide quantitative insight into oxide-scale morphology, this work focuses on real-time, in situ monitoring using infrared thermography. Previous studies on the same material have demonstrated that thermographic pixel-level features are physically linked to oxidation-related surface transformations, including oxide growth, emissivity changes, and spallation [19]. Future work will integrate the current thermographic framework with targeted SEM or spectroscopic analyses to establish quantitative relationships between surface proxies and oxide thickness. The potential of thermographic analysis in the steel industry is significant, as it offers a non-destructive method for monitoring and analysing surface processes by leveraging advanced computational models and machine learning techniques.

## 4. Conclusions

This study initially explores the detection and analysis of thermographic oxide scale using computer vision techniques. It also proposes a customised semantic segmentation architecture based on the SegNet Convolutional Neural Network (CNN), designed to learn the relevant features of the thermograms autonomously. The applied SegNet model was proven highly effective at accurately segmenting the samples. Oxide scale and background in the images were supported with advanced pre-processing and post-processing techniques to optimise detection performance. The adopted method demonstrates its adaptability. Similar analyses could expand the current database (2500 thermograms) to achieve greater accuracy and generalisation in predictions.

In developing the experimental methodology, the design of the steel specimen dimensions enabled control of the energy released by the Joule effect and ensured a constant temperature for each test condition. Once the target temperature between 150 °C and 700 °C was reached, it was maintained for 30 min in a still air environment, and thermographic images were acquired. The process was limited by the calibration recommendation of the thermal imaging camera and a constant emissivity coefficient. By analysing the pixel count per unit of time using machine learning techniques (across the entire domain for the various experimental cases), it was determined that the trend could be represented by a simple differential equation, as proposed in several studies on the kinetics of oxide layer thickening. As a result, pixel-level kinetic parameters were obtained, comparable in magnitude to those reported for oxide-layer thickening kinetics under ambient oxygen concentrations typical of normal conditions and a natural-convection airflow pattern. The CNN analysis technique was verified within the scope of this work, but not validated, using instrumentation to measure changes in oxide-layer thickness. Based on the observed results, it is assumed that, given the optical characteristics of reflection and radiation at the surface and the evolving state of the oxide, the counting technique is sensitive to pixelation changes arising from variations in chemical structure or physical relief, as well as the distribution of resistance to the passage of electric current that causes the temperature distribution in the recording section of the formation of the surface oxide scale. The explanation of the origin of slope changes in linear functions (splines) is left for future research, which will employ advanced structural analysis techniques. However, from the perspective of surface quality indicators for steel that is heat-treated by indirect methods, this work suggests implementing monitoring to determine the extent of change in the amount of oxide formed during a thermal process and its impact on subsequent processes. The success of this implementation depends on validating the behaviour of various case studies in which the steel composition varies and the oxygen concentration in the environment is measured or controlled.

## Figures and Tables

**Table 1:** Classification metrics for SegNet obtained on test sets.

Global Accuracy	Mean Accuracy	Mean IoU	Weight edIoU	Mean BFScore
96.40%	94.92%	83.29%	93.58%	81.37%

**Table 2:** Estimated surface magnitudes in Pixels^2^ of the white areas of the images in Figure 6 and associated colours.

Frame	Brightness, %	Surface Left, Pixels^2^	Surface Right, Pixels^2^	Colour
4	6	660	0	Pink
5	8	700	0	Light blue
6	10	1343 + 338 = 1681	0	Yellow
7	12	3170	0	Purple
8	14	4920	0	Orange
9	16	7815	533	Green
10	18	7936	1731 + 1906 = 3637	Dark blue
11	20	14,887	9539	Red
12	22	16,818	12,541	Black

## Data Availability

The experimental datasets generated and analysed during the current study are available in the Open Science Framework (OSF) repository: https://osf.io/2nvdm (accessed on 19 February 2026). The SegNet model was implemented using the standard MATLAB Deep Learning Toolbox, and the architectural and training details reported in Section 2.4 facilitate reproducibility. Additional raw data or trained model weights are available on request from the corresponding author. The following supporting information is available at: https://doi.org/10.5281/zenodo.17954461 (accessed on 19 February 2026).

## Supplementary Materials

The following supporting information can be downloaded at: https://www.mdpi.com/article/10.3390/ma19050920/s1, Figure S1: Optical macrographs of representative samples of AISI 1045 steel after cooling from high-temperature oxidation experiments, showing the spatial distribution of oxidation traces along the calibration section: (a) Experiment 1, (b) Experiment 2 and (c) Experiment 3, illustrating the variability in the location and degree of oxidation due to the Joule heating effect.

## Abbreviations

The following abbreviations are used in this manuscript:

CNN	Convolutional Neural Network
SEM	Scanning Electron Microscopy (SEM)
TEM	Transmission Electron Microscopy
XRD	X-ray diffraction
FIB	Focused Ion Beam
EDS	Energy-Dispersive X-ray Spectroscopy
AFM	Atomic Force Microscopy
SVM	Support Vector Machines
ANN	Artificial Neural Networks
AISI	American Iron and Steel Institute
IoU	Intersection over Union
BFScore	Boundary F1 Score
SGD	Stochastic Gradient Descent
RMSprop	Root Mean Square Propagation
CRF	Conditional Random Fields
