# Supplementary figures and images for: Kinetic Oxidation Analysis in AISI 1045 Steel Using Infrared Thermography and Convolutional Neural Networks
Source: Materials (Basel). 2026 Feb 27;19(5):920. doi: 10.3390/ma19050920 (PMC12985555; doi:10.3390/ma19050920)

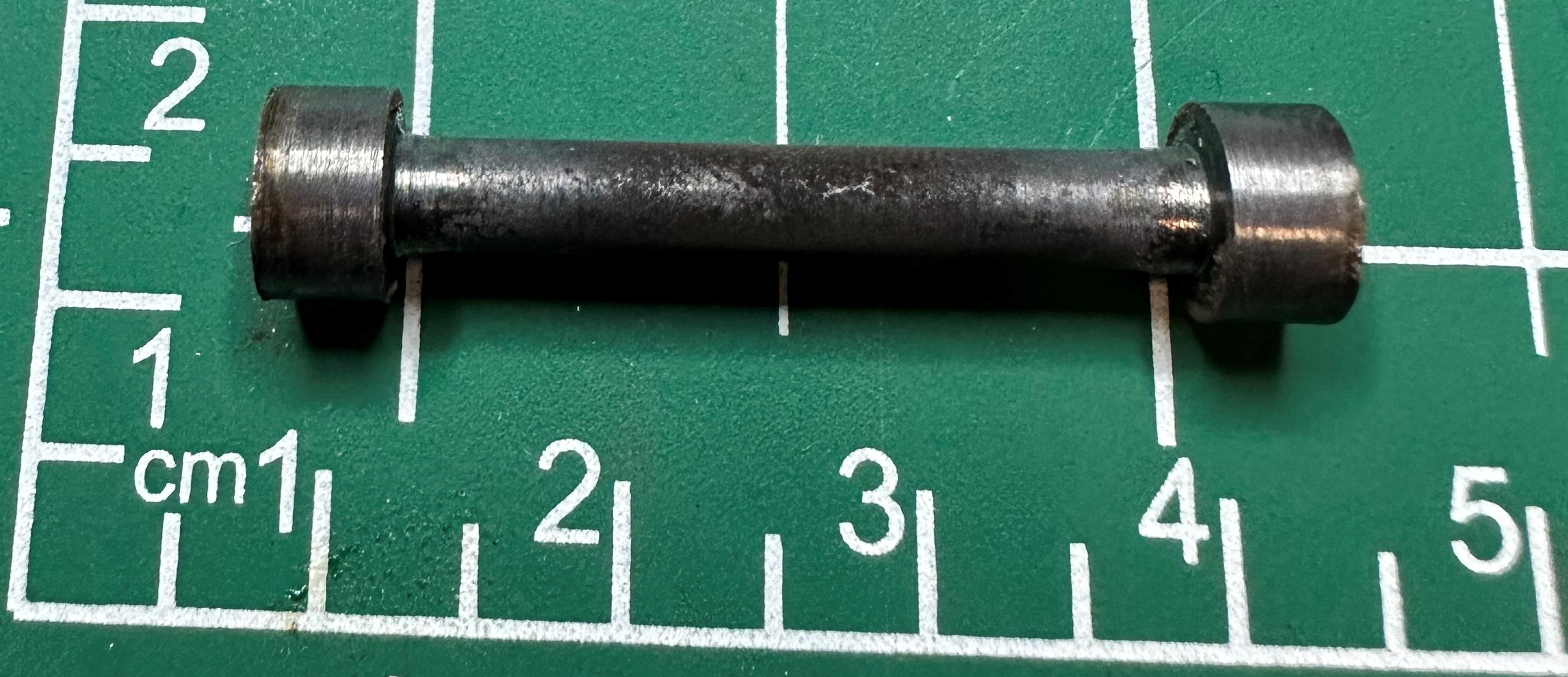

Supplement: Supplementary file 1 [file materials-19-00920-s001.zip › Figure_S1a.png]

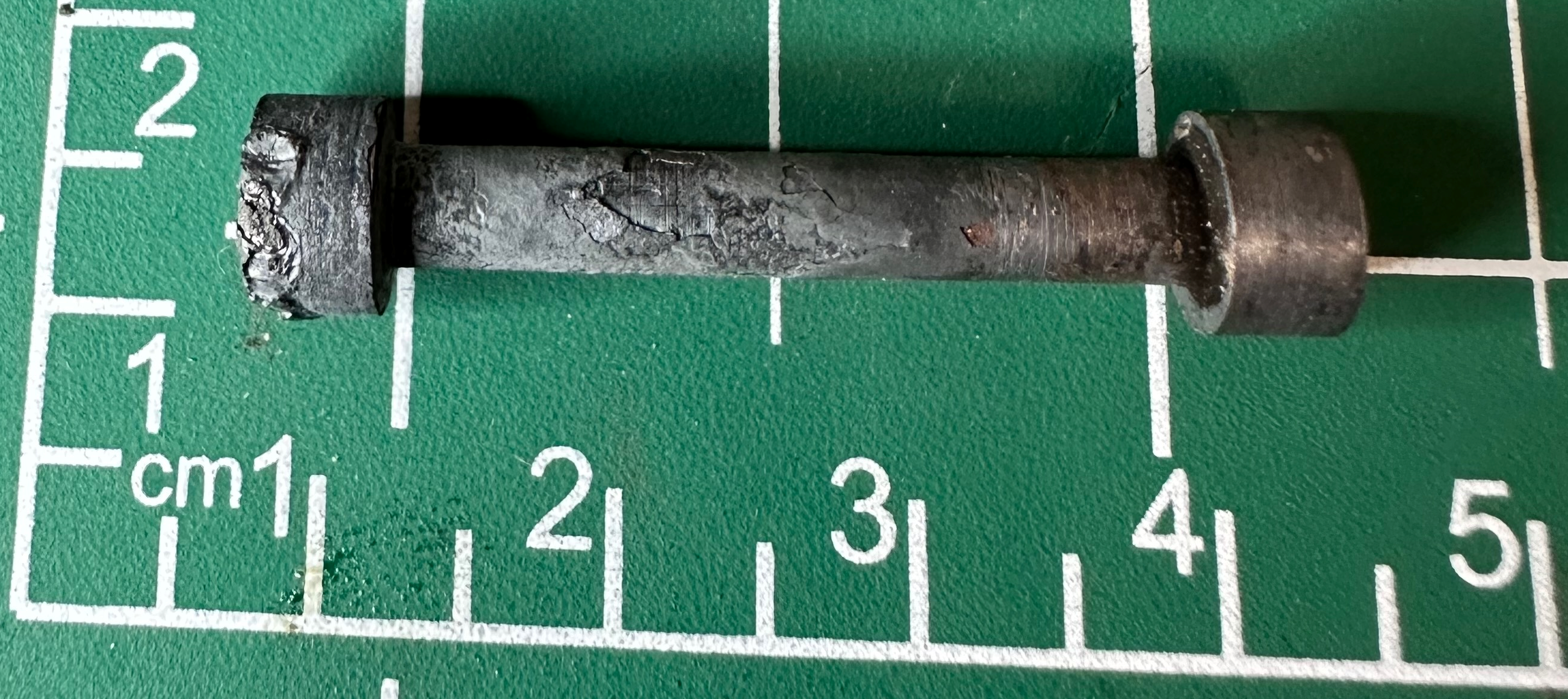

Supplement: Supplementary file 1 [file materials-19-00920-s001.zip › Figure_S1b.png]

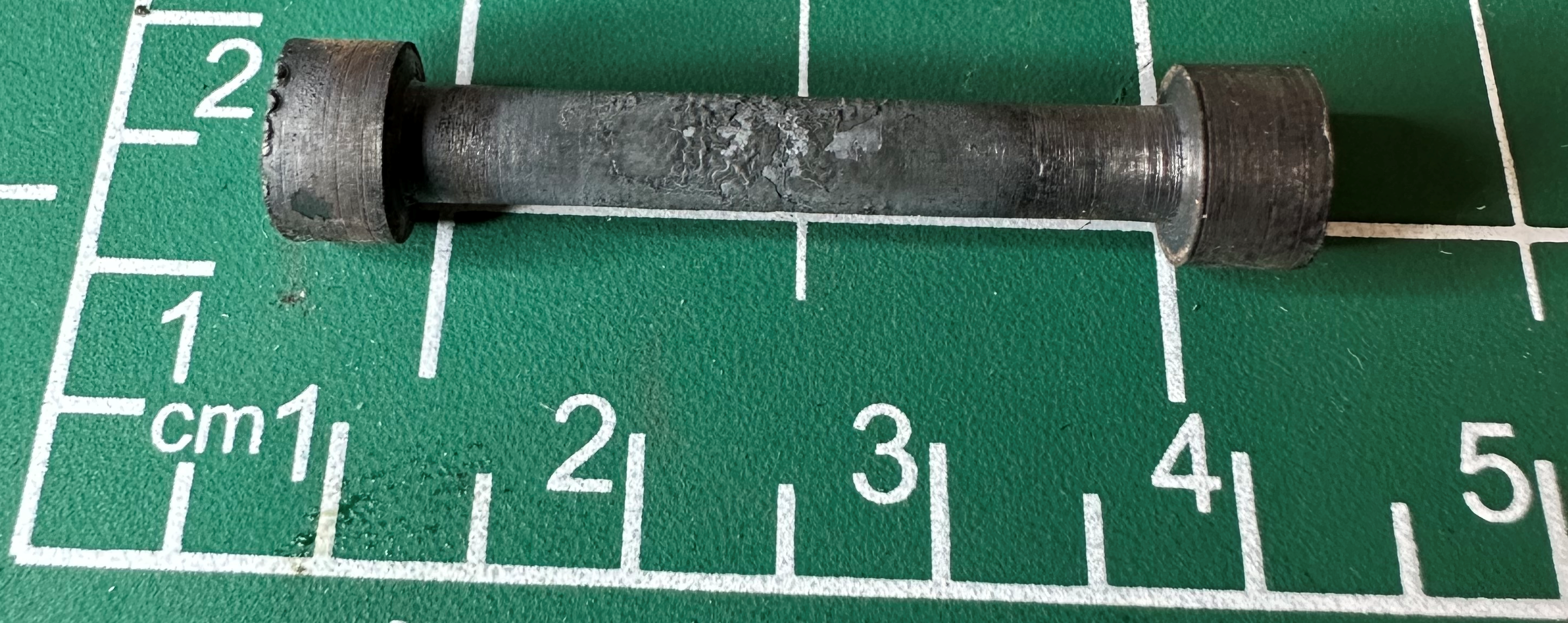

Supplement: Supplementary file 1 [file materials-19-00920-s001.zip › Figure_S1c.png]
